# Feasibility study on the effect and complications of intravenous therapy nursing intervention on patients with PICC catheterization

**DOI:** 10.1097/MD.0000000000047761

**Published:** 2026-05-01

**Authors:** Lanxin Guo, Wenshu Zhang, Tingting Hu, Haiying Xu

**Affiliations:** a PICC Clinic, Xiangyang Central Hospital, Affiliated Hospital of Hubei University of Arts and Science, Xiangyang, Hubei, China; b Oncology Department, Xiangyang Central Hospital, Affiliated Hospital of Hubei University of Arts and Science, Xiangyang, Hubei, China; c Nursing Department, Xiangyang Central Hospital, Affiliated Hospital of Hubei University of Arts and Science, Xiangyang, Hubei, China.

**Keywords:** compliance, complications, intravenous therapy specialist nursing, PICC, quality of life, self-management ability

## Abstract

The peripherally inserted central catheter (PICC) has been widely used in clinical practice, but there are also high risks in the use process. With the development of intravenous therapy technology, it is possible to reduce PICC related adverse events. This study aims to explore the effect of specialized nursing intervention of intravenous therapy in patients with PICC and the feasibility of preventing/reducing complications. Subjects 23 to 75 years treated with PICC catheterization concluded. A total of 96 subjects were assigned to a control group (n = 48), which performed routine care, a study group (n = 48), which received 2-week intravenous therapy specialist nursing therapy based on routine care. After 2 weeks for PICC nursing therapy, Chi square test analyses showed significant differences between routine care (75.00%) and intravenous therapy specialist nursing therapy (93.75%) in treatment compliance rate, as well as the scores of Cancer Patients PICC Sell management and the core scale of quality of life of cancer patients. Significantly lower incidence of complications for study group (8.33%) versus control group (27.08%). Intravenous therapy specialist nursing intervention has a significant effect on patients with PICC catheterization, which can improve patients’ treatment compliance, improve their quality of life, and effectively reduce the occurrence of related complications.

## 1. Introduction

Peripherally inserted central catheter (PICC) is a commonly used venous catheterization technique in clinic, which has the advantages of convenient operation, safety and reducing the pain of repeated puncture. It is the first choice for long-term infusion, especially for patients with malignant tumor chemotherapy.^[1,2]^ However, while PICC catheterization brings convenience to patients, there are also some problems, such as long wearing time, high risk of complications, affecting daily life, high nursing requirements, and routine clinical nursing has been unable to meet the development of PICC catheterization.^[3]^

With the development of vein therapy technology and medical concept, vein therapy specialist nurses have been born, and clinical practice has proved that the establishment of vein therapy specialist nursing team can improve the nursing quality of vein therapy and reduce the incidence of related adverse events.^[4-6]^ According to the survey, the treatment compliance of patients with PICC catheterization is generally not high, which not only affects the effect of treatment, but also reduces the quality of life of patients.

In view of this, this study took the patients with PICC catheterization as the research object to explore the application effect of intravenous therapy specialist nursing and the feasibility of reducing complications, so as to provide reference for the development and promotion of vein therapy specialist nursing work.

## 2. Materials and methods

### 2.1. Design and procedures

This study was approved by the Ethics Committee of Xiangyang Central Hospital. A retrospective study design was adopted. Clinical data of patients who underwent PICC catheterization at Xiangyang Central Hospital between September 2020 and September 2021 were retrospectively collected and analyzed. According to the nursing mode recorded in the medical records, patients were divided into 2 groups: the intravenous therapy specialist nursing group and the routine nursing group.

The routine nursing group received conventional PICC catheterization nursing care. The intravenous therapy specialist nursing group received specialist nursing care based on routine PICC nursing, including enhanced health education, daily observation, exercise guidance, and psychological nursing. Relevant data on treatment compliance, self-management ability, and quality of life were extracted from medical records and follow-up records, and the outcomes of the 2 groups were compared to evaluate the effects of different nursing strategies.

### 2.2. Setting and participants

The eligibility and exclusion criteria of patients were evaluated according to the inclusion situation. 18 to 75 years old; PICC catheterization; reading comprehension; complete clinical data. Exclusion criteria: failure of repeated catheterization; patients with 2 or more kinds of malignant tumors; patients with coagulation insufficiency; patients with severe mental illness. The ethics committee of the hospital approved this study

### 2.3. Procedure

#### 2.3.1. Control group

The control group carried out routine nursing intervention: explained the knowledge and precautions related to PICC catheter placement to the patients, regularly nursed/replaced the catheter, cleaned the skin at the puncture point, and instructed the patients and their families to maintain the catheter.

#### 2.3.2. Study group

The study group implemented intravenous therapy specialist nursing: a special nursing team for intravenous therapy was established. The members were nurses with infusion nurse qualification certificates and had more than 5 years of clinical nursing experience. Under the organization of the team leader, they learned the theoretical knowledge related to intravenous therapy every week, examined the practical ability of PICC, and summarized the problems and improvement methods in PICC work once a month. Health education: in addition to introducing their own disease-related knowledge to patients, the matters needing attention in PICC catheterization and the management of complications were sorted out into a pamphlet. Explained to patients/family members 1 by 1 to make them understand the necessity of PICC catheterization and the importance of catheter maintenance. Puncture nursing: before puncture, the position, range of motion, blood vessels and skin condition of the patient were carefully evaluated. After the best puncture point was found, the skin was routinely disinfected, punctured at an appropriate angle, and the action was kept gentle during operation. After the puncture was successful, the placement was checked immediately, and the catheter was fixed after there was no blood return. The skin and catheter around the puncture site were disinfected and cleaned before and after infusion every day. Daily observation and exercise nursing with tube: The possible situations of PICC catheterization were explained to patients in daily life, such as local pain, limb swelling, osmosis, return of blood, blockage or shedding of catheter, etc. The patient was told to report to the nurse as soon as the above situation was found. In addition, each time the catheter was maintained, the patient and their family members were explained the maintenance essentials and instructed to maintain the catheter by themselves. In view of problems such as catheter falling off, blocking and breaking that may occur during exercise, the patient was required to carefully check the stability of the catheter before exercised, covered and applied it, and the patient was instructed to reduce the range during exercise to avoid sweating at the puncture point, and immediately disinfected and cleaned the puncture point after exercise. Complication nursing: the catheterization of patients was closely watched, and the replacement frequency of application was increased. If redness, swelling or bleeding were found, ultraviolet radiation treatment or prophylactic use of antibiotics were given. During each intravenous drip, the infusion speed was adjusted according to the patient’s condition, and the liquid stimulation was reduced; at the same time, the tube was smoothed to avoid being discounted. Psychological nursing: a clean, hygienic, comfortable and warm ward environment was provided to patients, patients were communicated, patients’ psychological status was understood, and patients’ resistance to PICC catheterization was eliminated. A communication group of patients with PICC catheterization was set up, knowledge about PICC catheterization was sent every day, patients’ questions were answered, and patients were encouraged to share their experiences in nursing care of PICC catheters.

### 2.4. Measures

#### 2.4.1. Treatment compliance

Treatment compliance was assessed depending on the patient’s compliance with the doctor’s advice – Full compliance: the catheter was managed and maintained in full compliance with the doctor’s advice. Partial compliance: the operation was basically in accordance with the doctor’s orders, but occasionally nonstandard behavior. Disobey: in the vast majority of cases, catheter-related procedures were not completed in accordance with the doctor’s orders. Compliance rate = (full compliance + partial compliance)/total number of instances × 100%.

#### 2.4.2. Self-management ability

The self-management ability of the 2 groups was evaluated before and after intervention, and the evaluation tool was the Cancer Patients PICC Sell management (CPPSM).^[7]^ A total of 7 dimensions and 36 items were included, including daily life management (8 items), daily catheter observation (7 items), with tube movement management (4 items), maintenance compliance management (5 items), information acquisition (3 items), catheter abnormality management (4 items), and catheter management confidence (5 items). Each item is scored at Linker5 level (1–5 points). The total score ranged from 36 to 180. The higher the score, the better the self-management ability.

#### 2.4.3. Quality of life

Before and after intervention, the core scale of quality of life of cancer patients^[8]^ was used to evaluate the quality of life of the 2 groups. Physical function, emotional function, role function, cognitive function and social function were included in the scale. The total score of each field was 100. The higher the score, the better the quality of life of the patients.

#### 2.4.4. Complications

The incidence of complications during PICC catheterization in the 2 groups was counted. Phlebitis, catheter infection, bleeding at puncture point, ectopic or prolapsed catheter, and catheter blockage were common.

### 2.5. Data statistics

SPSS 24.0 software (IBM Corp., Armonk) was used for data analysis. The measurement data, concluding CPPSM scores, were in accordance with the normal distribution and had the same variance, which was expressed by (*x* ± *s*). The comparison between the 2 groups was performed by *t*-value test. The counting data was expressed in [n (%)], and *χ*^2^ tests were performed. The difference was statistically notable (*P* < .05). As this was a retrospective study, a formal sample size calculation was not conducted. The sample size was determined based on patient availability during the study period.

## 3. Results

### 3.1. Demographic characteristics at baseline

The mean age of the study group and the control group was 45.81 ± 10.33 years old and 44.95 ± 10.18 years old, and the mean course of disease was 2.59 ± 0.48 and 2.48 ± 0.43 respectively. There was no significant difference in the type of disease. As displayed in Table 1.

**Table 1:** Demographic characteristics at baseline.

Demographic and medical conditions	Study group(N = 48)	Control group (N = 48)	*χ*^2^/*t*	*P*
Age	45.81 ± 10.33	44.95 ± 10.18	0.411	.682
Gender (Man/Woman)	22/26	23/25	0.042	.841
BMI	21.51 ± 4.27	22.08 ± 3.97	0.68	.502
Course of disease	2.59 ± 0.48	2.48 ± 0.43	1.182	.240
Type of disease				
Breast cancer	14	13	0.266	.605
Gastric cancer	10	12		
Colorectal cancer	7	8		
Lung cancer	8	7		
Liver cancer	9	8		

BMI = body mass index.

### 3.2. Treatment compliance

**Figure 1.**
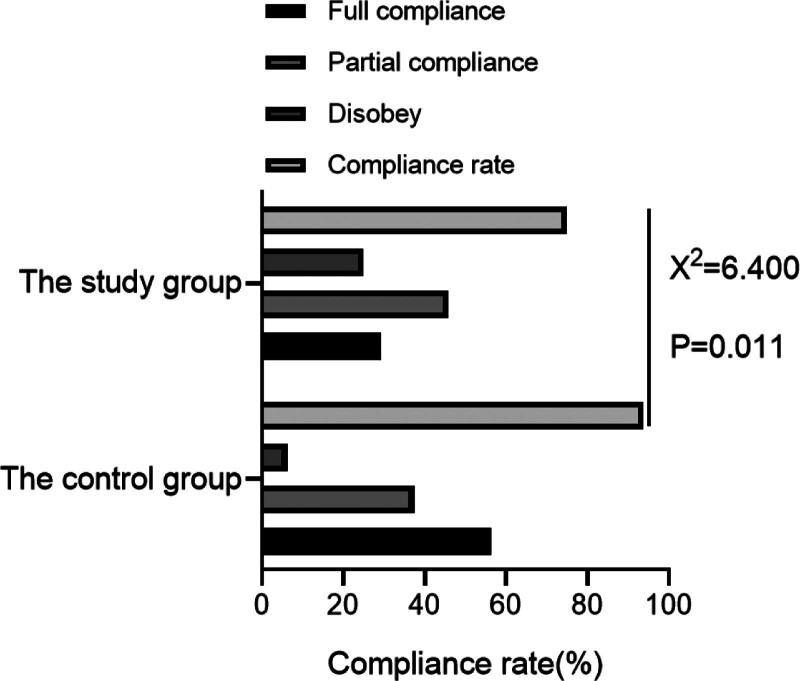
Compliance.

After intervention, versus the control group (75.00%), the treatment compliance rate of the study group (93.75%) was notably higher. As displayed in Table 2, Figure 1.

**Table 2:** Compliance [n (%)].

Groups	n	Full compliance	Partial compliance	Disobey	Compliance rate
The study group	48	27 (56.25)	18 (37.50)	3 (6.25)	45 (93.75)
The control group	48	14 (29.17)	22 (45.83)	12 (25.00)	36 (75.00)
*χ* ^2^					6.400
*P*					.011

### 3.3. Self-management ability

Before intervention, each dimension score and total score of CPPSM scale had no notable difference between the 2 groups (*P* > .05). After intervention, the scores of each dimension and the total score of the 2 groups were higher than those before intervention, and the increase of the study group was greater than that of the control group (*P* < .05). As displayed in Table 2.

**Figure 2.**
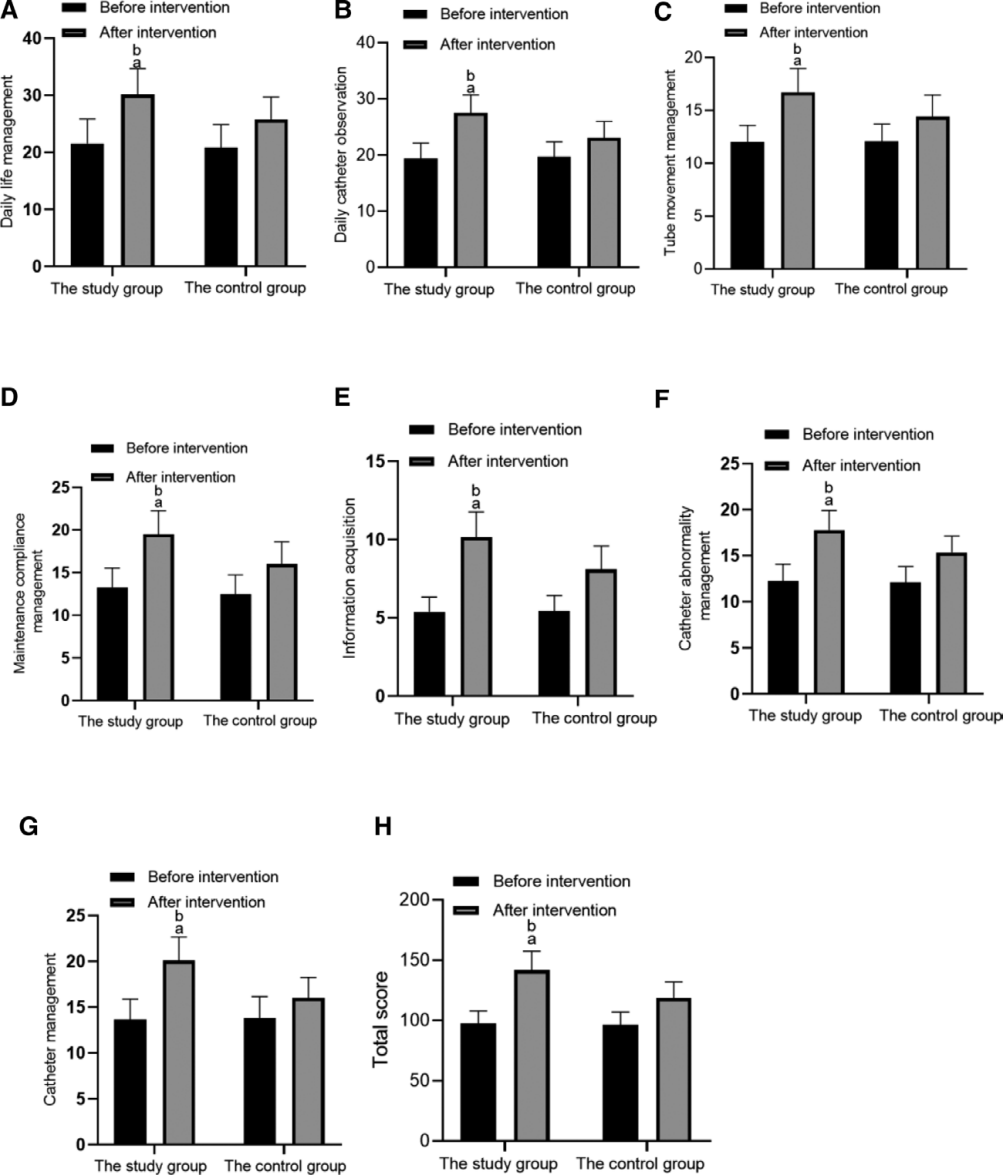
CPPSM scores. (A) Daily life management. (B) Daily catheter observation. (C) Tube movement management. (D) Maintenance compliance. (E) Information acquisition; (F) Catheter abnormality management. (G) Catheter management confidence. (H) Total score. CPPSM = Cancer Patients Peripherally Inserted Central Catheter Self-management.

Seven dimensions and 36 items are included, including daily life management (8 items), daily catheter observation (7 items), with tube movement management (4 items), maintenance compliance management (5 items), information acquisition (3 items), catheter abnormality management (4 items), and catheter management confidence (5 items). As displayed in Table 3, Figure 2.

**Table 3:** CPPSM scores (*x̄* ± *s*, score).

Dimensions	The study group (N = 48)		The control group (N = 48)	
	Before intervention	After intervention	Before intervention	After intervention
Daily life management	21.59 ± 4.26	30.17 ± 4.50*^,^**	20.84 ± 4.03	25.77 ± 3.94*
Daily catheter observation	19.41 ± 2.78	27.55 ± 3.16*^,^**	19.75 ± 2.64	23.10 ± 2.89*
Tube movement management	12.05 ± 1.54	16.73 ± 2.24*^,^**	12.11 ± 1.62	14.43 ± 2.05*
Maintenance compliance management	13.21 ± 2.35	19.50 ± 2.75*^,^**	12.46 ± 2.28	16.01 ± 2.60*
Information acquisition	5.38 ± 0.95	10.15 ± 1.62*^,^**	5.45 ± 0.98	8.11 ± 1.48*
Catheter abnormality management	12.25 ± 1.84	17.78 ± 2.15*^,^**	12.14 ± 1.70	15.36 ± 1.79*
Catheter management confidence	13.67 ± 2.21	20.10 ± 2.58*^,^**	13.80 ± 2.37	16.04 ± 2.23*
Total score	97.56 ± 10.34	141.98 ± 15.60*^,^**	96.55 ± 10.61	118.82 ± 13.24*

Compared with the same group before intervention.

CPPSM = Cancer Patients Peripherally Inserted Central Catheter Self-management.

* P < .05; compared with the control group after intervention.

** P < .05.

### 3.4. Quality of life

**Figure 3.**
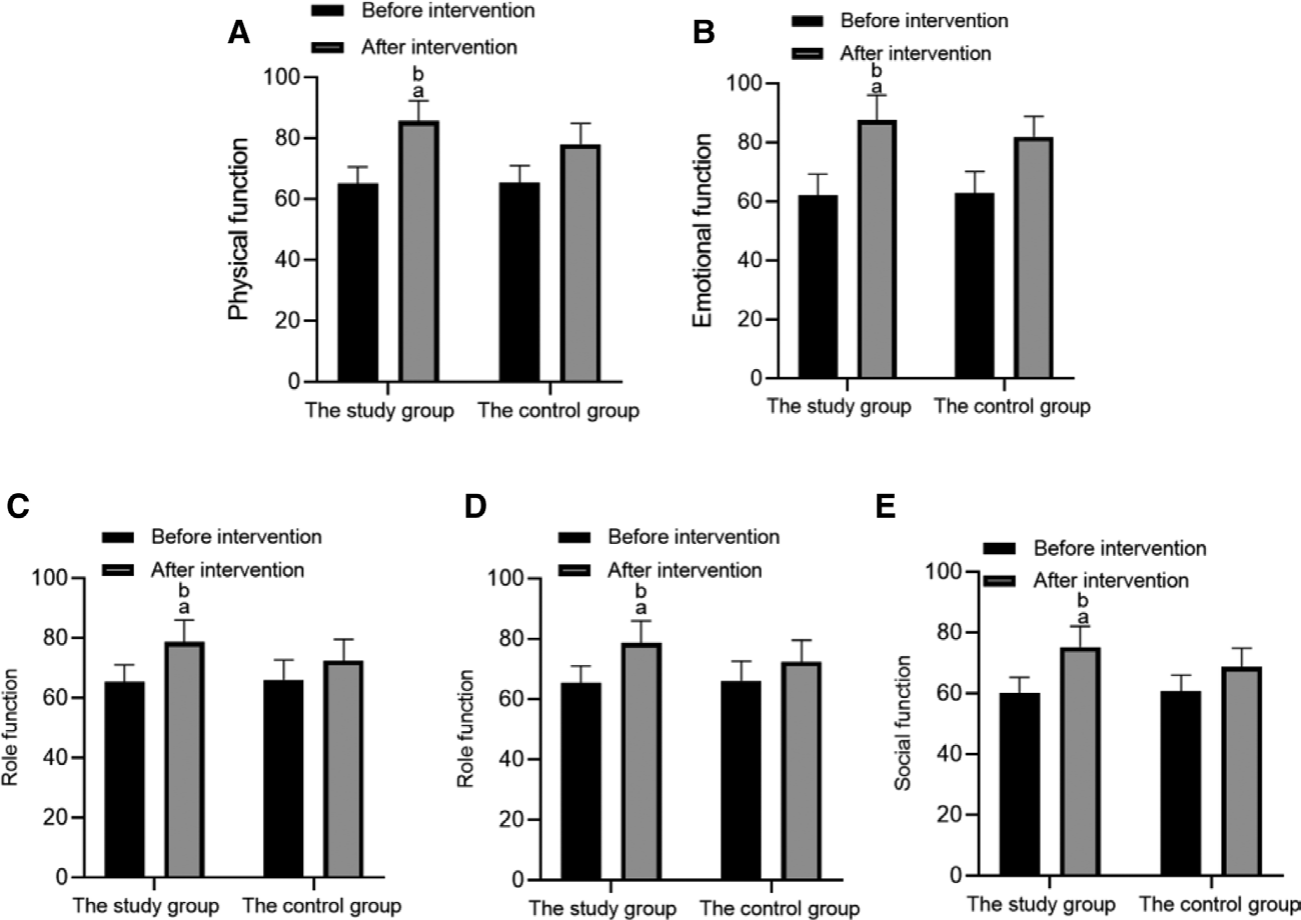
The scores of quality of life scale between the 2 groups before and after intervention. (A) Physical function; (B) emotional function; (C) role function; (D) cognitive function; (E) social function.

Before intervention, there was no difference in the scores of physical function, emotional function, role function, cognitive function and social function between the 2 groups (*P* > .05). After intervention, the scores of all fields in both groups increased (*P* < .05), and the scores in all fields in the study group were significantly higher than those in the control group (*P* < .05). As shown in Table 4, Figure 3.

**Table 4:** The scores of quality of life scale between the 2 groups before and after intervention (*x̄* ± *s*, score).

Fields	The study group (N = 48)		The control group (N = 48)	
	Before intervention	After intervention	Before intervention	After intervention
Physical function	65.26 ± 5.33	85.72 ± 6.67*^,^**	65.58 ± 5.49	78.14 ± 6.81*
Emotional function	62.15 ± 7.21	87.65 ± 8.44*^,^**	63.02 ± 7.18	81.92 ± 7.04*
Role function	65.42 ± 5.67	78.73 ± 7.28*^,^**	66.10 ± 6.61	72.47 ± 7.13*
Cognitive function	68.14 ± 5.37	83.80 ± 7.50*^,^**	68.55 ± 5.82	75.09 ± 6.42*
Social function	60.11 ± 5.24	75.32 ± 6.79*^,^**	60.72 ± 5.37	68.75 ± 6.18*

Compared with the same group before intervention.

* P < .05; compared with the control group after intervention.

** P < .05.

### 3.5. Complications

The incidence of complications in the study group and the control group was 8.33% and 27.08% respectively, the difference was significant. As shown in Table 5.

**Table 5:** Comparison of the incidence of complications between 2 groups, n (%).

Groups	n	Phlebitis	Catheter infection	Puncture point bleeding	Ectopic or prolapsed catheter	Catheter blockage	Total occurrence
The study group	48	1 (2.08)	1 (2.08)	0 (0.00)	2 (4.17)	0 (0.00)	4 (8.33)
The control group	48	3 (6.25)	2 (4.17)	3 (6.25)	4 (10.43)	1 (2.08)	13 (27.08)
*χ* ^2^							5.790
*P*							.016

## 4. Discussion

Intravenous therapy is the most widely used nursing technology in clinic. With the development of medical technology, its nursing content is not only limited to infusion operation, but also involves medicine, infection management, nutrition management and other disciplines.^[9]^ For this reason, the American Society of intravenous Infusion Nursing put forward the concept of intravenous therapy specialist nurses, thus greatly improving the quality of intravenous therapy nursing.^[1]^ The training of intravenous therapy specialist nurses in China is later than that in foreign countries. at present, their nursing work has not been unified in content and form. Therefore, this study deeply discusses the application effect of intravenous therapy specialist nursing in clinical practice. PICC refers to an intravenous infusion technique in which the tip of a patient’s peripheral vein is intubated so that the tip is located in the large vein near the heart.^[10]^ According to the study,^[11]^ PICC is easy to operate, can avoid repeated operation, protects the arm vein, and is suitable for long-term intravenous therapy, chemotherapy, parenteral nutrition and so on. The results of this study showed that, compared with the control group, the treatment compliance of the study group is higher, indicating that intravenous therapy specialist nursing can improve the treatment compliance of patients with PICC catheterization. Previous investigations have shown that affecting daily life, limb functional exercise and out-of-hospital catheter maintenance are the main factors affecting cancer patients receiving PICC catheterization.^[12]^ The establishment of intravenous therapy specialist nursing team brings sense of security and trust to patients professionally, which can increase patients’ acceptance of PICC and improve treatment compliance. In addition, the targeted health education and nursing guidance of intravenous therapy specialist nurses can also improve patients’ awareness of PICC catheterization, help patients master the key points of catheter nursing and improve their enthusiasm for treatment.^[13]^ Wang et al^[14]^ have shown that the combined intervention of psychological nursing and health education can improve the compliance of chemotherapy patients with PICC catheterization.

Self-management ability is an important indicator reflecting the nursing quality of PICC catheterization.^[15]^ In this study, the scores of all dimensions of CPPSM scale in the study group after intervention were significantly higher than those before intervention and those in the control group, indicating that intravenous therapy can improve the self-management ability of patients with PICC catheterization, which is conducive to the promotion of PICC catheterization. Analysis reason: On the one hand, after the training of theoretical knowledge and skills, the nursing ability of nurses has been greatly improved, which indirectly affects the self-management ability of patients. On the other hand, the education of PICC catheterization related knowledge and the guidance of catheter maintenance helped patients establish catheter management beliefs and improve their catheter. In addition, WeChat communication groups can also play a role in urging and assisting patients to improve their self-management ability.^[16-18]^ Previous studies^[19]^ found that the self-management ability of patients with PICC catheterization is lower than that of other patients, which is related to patients’ lack of relevant knowledge, and targeted health education and guidance can improve patients’ self-management ability. This study also shows that intravenous therapy specialist nursing can effectively improve the quality of life of patients. Patients with PICC catheterization need to live with the tube for a long time, which may affect daily activities such as bathing and sports. The improvement of patients’ self-management ability can greatly improve their quality of life.^[20]^

Compared with previous studies, our findings further support the role of structured intravenous therapy specialist nursing in improving patient compliance and reducing PICC-related complications. Based on our results, it is recommended to promote standardized training programs for intravenous therapy specialist nurses and integrate continuous nursing models into routine clinical practice.

This study has several limitations. First, the sample size was relatively small, and the study was conducted at a single center, which may limit the generalizability of the findings. Second, blinding of participants and nursing staff was not feasible due to the nature of the intervention, which may introduce performance and social expectation bias. Third, the follow-up period was relatively short, and long-term outcomes could not be evaluated. Future multicenter studies with larger sample sizes and longer follow-up periods are warranted to confirm these findings.

PICC catheterization is an invasive operation, and long-term retention in the body will lead to phlebitis, bleeding at the puncture point, catheter infection, prolapse and other complications.^[21]^ In this study, the incidence of complications in the study group was 8.33%, significantly lower than that in the control group (27.08%), indicating that special nursing of intravenous therapy can reduce the incidence of complications in PICC catheterization. This is consistent with the results of previous similar study,^[22]^ indicating that it is feasible to use intravenous therapy specialist nursing to prevent/ reduce the incidence of complications related to PICC catheterization.

To sum up, intravenous specialist nursing for patients with PICC catheterization is feasible, which can not only prevent/reduce the occurrence of complications, but also improve the quality of life of patients, which is worth popularizing.

## Author contributions

**Conceptualization:** Lanxin Guo, Wenshu Zhang, Tingting Hu, Haiying Xu.

**Data curation:** Lanxin Guo, Wenshu Zhang, Tingting Hu, Haiying Xu.

**Formal analysis:** Lanxin Guo, Wenshu Zhang, Tingting Hu, Haiying Xu.

**Funding acquisition:** Haiying Xu.

**Investigation:** Haiying Xu.

**Writing – original draft:** Haiying Xu

**Writing – review & editing:** Haiying Xu.

## References

[1] Sherwood G Nickel B. Integrating quality and safety competencies to improve outcomes: application in infusion therapy practice. J Infus Nurs. 2017;40:116–22. 28248812 10.1097/NAN.0000000000000210

[2] Gorski LA. The 2016 infusion therapy standards of practice. Home Healthc Now. 2017;35:10–8. 27922994 10.1097/NHH.0000000000000481

[3] Parás-Bravo P Paz-Zulueta M Santibañez M. Living with a peripherally inserted central catheter: the perspective of cancer outpatients-a qualitative study. Support Care Cancer. 2018;26:441–9. 28707169 10.1007/s00520-017-3815-4 PMC5752737

[4] Ferlay J Colombet M Soerjomataram I. Estimating the global cancer incidence and mortality in 2018: GLOBOCAN sosurces and methods. Int J Cancer. 2019;144:1941–53. 30350310 10.1002/ijc.31937

[5] Li Y Wan M Luo X. The impact of informing diagnosis on quality of life in patients with cancer: a protocol of systematic review and meta-analysis. Medicine (Baltim). 2018;97:e12320. 10.1097/MD.0000000000012320 PMC6155951 30212976

[6] Zarkesh MR Haghjoo M. Neonatal cardiac tamponade, a life-threatening complication secondary to peripherally inserted central catheter: a case report. J Med Case Rep. 2022;16:305. 35902974 10.1186/s13256-022-03506-4 PMC9335958

[7] Piredda M Sguanci M De Maria M. Nurses’ evidence-based knowledge and self-efficacy in venous access device insertion and management: development and validation of a questionnaire. Nurs Open. 2024;11:e2177. 38967938 10.1002/nop2.2177 PMC11225607

[8] Sacomori C Lorca LA Martinez-Mardones M. A randomized clinical trial to assess the effectiveness of pre- and post-surgical pelvic floor physiotherapy for bowel symptoms, pelvic floor function, and quality of life of patients with rectal cancer: CARRET protocol. Trials. 2021;22:448. 34256795 10.1186/s13063-021-05396-1 PMC8276537

[9] Ding N Peng H Zhao W. Effects of Peripherally inserted Central Catheter (PICC) materials and designs on reduction of PICC-related complications: a systematic review and meta-analysis. Int Wound J. 2024;21:e14468. 38050652 10.1111/iwj.14468 PMC10898378

[10] Chan RJ Northfield S Larsen E. Central venous Access device SeCurement And Dressing Effectiveness for peripherally inserted central catheters in adult acute hospital patients (CASCADE): a pilot randomised controlled trial. Trials. 2017;18:458. 28978332 10.1186/s13063-017-2207-x PMC5628427

[11] Toro A Schembari E Mattone E Di Carlo I. Which is better for patients with breast cancer: Totally Implanted Vascular Access Devices (TIVAD) or Peripherally Inserted Central Catheter (PICC)? World J Surg. 2020;44:1004–5. 31541273 10.1007/s00268-019-05196-4

[12] Burbridge B Lim H Dwernychuk L. Comparison of the quality of life of patients with breast or colon cancer with an arm vein port (TIVAD) versus a Peripherally Inserted Central Catheter (PICC). Curr Oncol. 2021;28:1495–506. 33918869 10.3390/curroncol28020141 PMC8167661

[13] Zhu W Liu J Qian H Wu Y Xu CM. Application of continuous nursing intervention for patients with PICC catheterization undergoing tumor chemotherapy. Am J Transl Res. 2021;13:7207–13. 34306483 PMC8290827

[14] Wang Y Li J Wang Y. The influential factors and intervention strategies that engage malignant cancer patients in health-promoting behaviors during PICC line maintenance. Am J Transl Res. 2021;13:5208–15. 34150110 PMC8205709

[15] Mielke D Wittig A Teichgräber U. Peripherally inserted central venous catheter (PICC) in outpatient and inpatient oncological treatment. Support Care Cancer. 2020;28:4753–60. 31970514 10.1007/s00520-019-05276-0 PMC7447660

[16] Chen N Yang Q Li YF Guo Q Huang Y Peng JL. Cost-utility analysis of different venous access devices in breast cancer patients: a decision-based analysis model. BMC Health Serv Res. 2023;23:497. 37194042 10.1186/s12913-023-09517-1 PMC10190063

[17] Fan F Zou Y Zhang S. Rivaroxaban in the treatment of PICC-associated upper extremity venous thrombosis. Clin Ther. 2017;39:1882–8. 28823518 10.1016/j.clinthera.2017.07.041

[18] Konstantinou EA Mariolis Sapsakos TD Katsoulas TA Velecheris D Tsitsimelis D Bonatsos G. Persistent left superior vena cava leads to catheter malposition during PICC Port placement. J Vasc Access. 2016;17:e29–31. 26797899 10.5301/jva.5000498

[19] Zhang N Xu Y Lu Q. Exploring the behavioral intentions of PICC-related thrombosis prevention in breast cancer patients undergoing chemotherapy: a qualitative study based on theory of planned behavior. Support Care Cancer. 2024;32:635. 39235516 10.1007/s00520-024-08827-2

[20] Normann CO Opheim R Andreassen BK Bernklev T Haug ES. Health-related quality-of-life after radical cystectomy among Norwegian men and women compared to the general population. Scand J Urol. 2020;54:181–7. 32343159 10.1080/21681805.2020.1754906

[21] Leung TK Lee CM Tai CJ Liang YL Lin CC. A retrospective study on the long-term placement of peripherally inserted central catheters and the importance of nursing care and education. Cancer Nurs. 2011;34:E25–30. 20885304 10.1097/NCC.0b013e3181f1ad6f

[22] Patankar S. Combining infrared vein visualization and ultrasound guidance for central line placement in difficult venous access patients: a technical report. Cureus. 2025;17:e83264. 40453300 10.7759/cureus.83264 PMC12124818

